# Personality traits have an effect on pain-related psychological variables in patients with chronic low back pain

**DOI:** 10.1371/journal.pone.0354827

**Published:** 2026-07-31

**Authors:** Kazuhiro Hayashi, Kenji Miki, Yui Fukushima, Mayuu Iwai, Yukiko Shiro, Tomoko Tetsunaga, Toshifumi Takasusuki, Masako Hosoi, Masao Yukioka, Seiji Okada

**Affiliations:** 1 Human Health Sciences, Graduate School of Medicine, Kyoto University, Kyoto, Japan; 2 Japan Pain Foundation, Tokyo, Japan; 3 Center for pain management, Hayaishi Hospital, Osaka, Japan; 4 Faculty of Health Science, Osaka Yukioka College of Health Science, Ibaraki, Japan; 5 Department of Physical Therapy, Faculty of Rehabilitation Sciences, Nagoya Gakuin University, Nagoya, Japan; 6 Faculty of Human Life Sciences, Notre Dame Seishin University, Okayama, Japan; 7 Department of Anesthesiology, Dokkyo Medical University, Mibu, Japan; 8 Department of Psychosomatic Medicine and Multidisciplinary Pain Center, Kyushu University Hospital, Fukuoka, Japan; 9 Department of Orthopaedic Surgery, Graduate School of Medicine, Osaka University, Suita, Japan; Karolinska Institutet, SWEDEN

## Abstract

**Introduction:**

An association between personality factors and pain experience has been investigated. However, the direct contributions of personality traits by the Maudsley Personality Inventory to clinical pain outcomes in patients with chronic pain remain unclear. The Maudsley Personality Inventory is a self-report questionnaire used to assess extroversion, neurotic tendencies, and lying or exhibitionistic tendencies. The present study aimed to investigate whether personality traits influenced with the pain-related psychological variables in patients with chronic secondary low back pain (CLBP).

**Methods:**

One hundred fifteen consecutive outpatients with CLBP were included. Personality traits were measured by using the Maudsley Personality Inventory to assess extroversion, neurotic tendencies, and lying or exhibitionistic tendencies. The association of Maudsley neurotic tendency score and pain-related variables was analyzed and then performed a path analysis. Eleven patients (9%) were the biased persons with frequent lying.

**Results:**

The pain-related variables showed no significant differences between the persons with frequent lying and those with normal. The Maudsley neurotic tendency score was significantly associated with Pain Catastrophizing Scale, Hospital Anxiety and Depression Scale, Pain Self-Efficacy Questionnaire, Athens Insomnia Scale, and EuroQol-5 Dimensions-3 level. The unstandardized coefficients for Maudsley neurotic tendency score were small, compared to pain-Numerical Rating Scale in each outcome.

**Conclusions:**

These results suggest the presence of neurotic tendency could have an impact on the health outcomes in patients with CLBP, but the impact was small.

## Introduction

The lifetime prevalence of low back pain has been reported to be as high as 84%, and estimates suggest that approximately 23% of individuals experience chronic low back pain, with 11–12% of the population being disabled by low back pain across diverse populations [1]. Chronic pain is defined as pain that persists or recurs for longer than 3 months [2]. Chronic pain interferes with daily functioning, and is often accompanied by emotional distress [2]. A bio-psycho-social model is recommended to inform assessment and management, given the associations between behavioral, psychological, and social factors and the future persistence of pain and disability [3]. Pain-related psychological disturbances are widely assessed using self-reported questionnaires [4].

Psychological disturbance is influenced by personality trait [5,6]. Personality traits are enduring patterns of perceiving, relating to, and thinking about oneself and the environment, and are exhibited across a wide range of social and personal contexts [7]. Neuroticism is strongly associated with psychological disturbance, whereas extraversion shows independent effect [5,6]. Since the 19th century, an association between personality factors and the pain experience has been investigated [8]. A recent review concluded that personality traits are associated with experimental pain in healthy individuals [9] and clinical pain outcomes in patients with musculoskeletal pain [10]. Neuroticism and extraversion are associated with health outcomes in patients with chronic pain in the Western countries [11–13], while not adequately investigated in non-Western country. Most studies assess personality traits using the Big-Five trait model, which includes extraversion, agreeableness, conscientiousness, neuroticism, and openness to experience [9,10]. The inventory does not typically include items assessing lying or biased responding [5,6]. When individuals are asked to rate themselves across personality dimensions, they may exaggerate their positive qualities and downplay their negative qualities [14]. The big five are not discrete, independent dimensions but rather represent partially overlapping and increasingly complex groups of facets [15,16]. Another, most studies are reported in the Western countries, even though Asians scored higher on neuroticism, and lower on extraversion, conscientiousness, and openness [17].

Then, a tool for alternative personality traits, the Maudsley Personality Inventory, includes a scale comprising 20 items designed to test lying [18]. It is a self-report questionnaire used to assess extroversion, neurotic tendencies, and lying or exhibitionistic tendencies, and is associated with psychological functioning. The items of Maudsley were selected to minimize correlation between the neurotic tendency and extraversion scales [19]. Shiomi 1978 first suggested that the Maudsley neurotic tendency score (N-score) and extraversion score (E-score) are associated with experimental pain in healthy individuals [20]. Later, Kasai 2017 suggested that Maudsley lye score (L-score) is significantly higher among outpatients experiencing a high degree of pain [21]. Personality traits, as well as pain intensity, have been shown to influence pain-related psychological outcomes [22]. However, the direct contributions of personality traits by the Maudsley Personality Inventory to clinical pain outcomes in patients with chronic pain remain unclear.

The present study aimed to investigate whether personality traits influence pain-related psychological variables in patients with chronic secondary low back pain (CLBP).

## Methods

### Participants

Participants were recruited consecutively from outpatient clinic in Japan between 17/01/2025 and 19/12/2025. The patients were eligible if they (1) were older than 18 years of age, (2) were diagnosed with chronic secondary pain in low back due to trauma or surgery, and (3) had persistent low back pain for at least 6 months prior to enrollment in the study. Exclusion criteria were: (1) unwillingness to participate, (2) pain related to tumors or presence of neurological symptoms, (3) surgery within the previous 3 months, (4) use of medications associated with dementia, and (5) were poor Japanese language comprehension. All inclusion and exclusion criteria were assessed by the referring physicians.

This cross-sectional study was approved by the Ethics Committee of Hayaishi Hospital (R070110-01), and all study participants provided written informed consent. All experiments were registered (UMIN000056708; Registration, Jan 14, 2025). All methods were carried out in accordance with relevant guidelines and regulations, in accordance with the Declaration of Helsinki.

### Measures

Demographic data, including age, sex, height, weight, body mass index, marital status, exercise habits, welfare recipient status, occupation, and education were collected during the participants’ first visit to the clinic. Personality traits were assessed using the Japanese version of the Maudsley Personality Inventory [19,23]. The Maudsley E-score can ranges from 0 to 48, with higher scores indicating greater extroversion (normal average, 21–31). The Maudsley N-score ranges from 0 to 48, with higher scores indicating greater neurotic tendencies (normal average, 19–28). The Maudsley L-score ranges from 0 to 40, with higher scores reflecting higher levels of lying or exhibitionistic tendencies (normal average, 0–25).

Pain related variables were assessed using the following questionnaires: the Pain-Numerical Rating Scale (NRS), Pain Catastrophizing Scale (PCS), Hospital Anxiety and Depression Scale (HADS), Pain Disability Assessment Scale (PDAS), Pain Self-Efficacy Questionnaire (PSEQ), Athens Insomnia Scale, 25-question Geriatric Locomotive Function Scale (Locomo-25), and EuroQol-5 Dimensions-3 level (EQ-5D-3L). All questionnaires included translated versions in Japanese and were analyzed as continuous variables.

Pain intensity was measured using the 0 − 10 NRS [24]. The pain-NRS scale, ranging from 0 to 10, was used to indicate the average level of pain experienced during the day, with 0 representing “no pain” and 10 representing the “worst pain imaginable.”

The PCS score consists of 13 items, with participants indicating how frequently they experience each cognition or emotion [25,26]. The total PCS score ranges from 0 to 52, with higher scores reflecting greater levels of catastrophizing.

The HADS was designed to assess two separate dimensions of anxiety and depression [27,28]. The HADS score consists of 14 items, with the anxiety (HADS-Anxiety) and depression (HADS-Depression) subscales each containing 7 items. Responses are rated on a four-point scale, ranging from 0 (absence of symptoms) to 3 (maximum symptoms), resulting in possible subscale scores from 0 to 21.

The PDAS score assesses the extent to which chronic pain interferes with various daily activities over the past week [29]. The PDAS comprises 20 items covering a broad range of daily activities, with respondents indicating the degree of interference caused by pain. Total scores range from 0 to 60, with higher scores reflecting greater levels of pain interference.

The PSEQ score assesses the self-efficacy beliefs despite pain [30,31]. The PSEQ comprises 10 items reflecting pain self efficacy. It regards self-efficacy in physical functioning, social interactions and engagement in valued activities. Total scores range from 0 to 60, with lower scores indicating lower levels of self-efficacy.

The Athens Insomnia Scale score assesses the severity of insomnia symptoms [32,33]. Total scores range from 0 to 24, with higher scores indicating greater insomnia severity.

The Locomo-25 score assesses the declines in mobility functions due to locomotive organ impairment [34]. It evaluates impairments in the musculoskeletal system resulting in pain, limited range of motion, muscle weakness, or balance problems. Total scores range from 0 to 100, with higher scores indicating greater difficulties and disabilities in daily activities related to locomotive function.

The EQ-5D-3L scores assess subjective health-related quality of life (QOL) [35,36]. The instrument evaluates health across five dimensions: mobility, self-care, usual activities, pain/discomfort, and anxiety/depression. Each dimension is assessed with a single question offering three possible responses: no problems, some problems, or serious health problems. EQ-5D-3L scores range from −0.111 to 1.000, with negative scores indicating health states considered worse than death, 0 representing death, and 1.000 representing full health.

### Statistical analysis

All continuous data are expressed as means and standard deviations. The normality of continuous variables was evaluated using the Shapiro-Wilk test. Depending on the data distribution, groups classified according to the Maudsley E-, N-, or L-scores were compared using either unpaired t-tests or Mann-Whitney U tests. Categorical variables are represented as counts and percentage, and were analyzed using the χ2 test. Correlations among continuous variables were assessed using the Pearson correlation coefficient.

Path analysis was conducted to examine relationships among variables. Pain-related psychological variables that showed significant and moderate correlation with the Maudsley N-score (p-value, < 0.05; and correlation coefficient, > 0.3) were included as primary outcomes in the subsequent path analysis. The Maudsley N-score and pain-NRS were included as predictors. Unstandardized coefficient, standard errors, and 95% confidence interval were calculated. The unstandardized coefficient represented the expected change in the outcome variable for each one-unit increase in the predictor variable. Model fit was assessed using the goodness of fit index (GFI) > 0.95, adjusted goodness of fit index (AGFI) > 0.95, comparative fit index (CFI) > 0.95, Tucker-Lewis index (TLI) > 0.95, and root mean square error of approximation (RMSEA) ≤ 0.05 [37]. All analyses were performed using JMP Pro 17 (version 17.0.0, SAS Institute Inc., Cary, NC, USA). A p value of < 0.05 was considered statistically significant.

The sample size was calculated using the G*Power software (version 3.1.9.2; Franz Faul, Kiel University, Kiel, Germany). Based on an effect size of 0.26 [11–13] the minimum number of subjects was estimated to be 111 for an α-level of 0.05, and a power (1 – β) of 0.80. Because we expected that the data from persons with frequent lying could be outliers [38], 115 patients with CLBP were recruited.

## Results

Patient characteristics are shown in **Table 1**. The mean age of the patients was 54.9 years. Of the 115 patients, 73 (63%) were women. The mean pain-NRS value was 5.5. Eleven patients (9%) were the biased persons with frequent lying (Maudsley L-score, > 25 points). The pain-related variables showed no significant differences between the persons with frequent lying and those with normal, except for PDAS score. The patients with the neurotic tendency (Maudsley N-score, > 28 points) consistently showed the introversion trait (Maudsley E-score, < 21 points) (S1A Table). The Maudsley subscores showed small or moderate correlations with each other (S1B Table).

**Table 1:** Patient characteristics and comparison of data between patients with frequent lying and those with normal (Maudsley L-score) (n = 115).

	Overall (n = 115)	Low L-score ≤ 25 points (n = 104)	High L-score > 25 points (n = 11)	p-value
Age, year	54.9 (15.3)	54.7 (15.2)	56.8 (16.0)	0.672
Women, n (%)	73 (63%)	65 (62%)	8 (72%)	0.503
Height, cm	160.3 (8.4)	160.7 (8.5)	156.7 (5.9)	0.131
Weight, kg	59.3 (13.4)	59.6 (13.7)	56.0 (10.9)	0.409
Body mass index, kg/m^2^	22.9 (4.2)	22.9 (4.3)	22.7 (3.9)	0.894
Married, n (%)	71 (61%)	66 (63%)	5 (45%)	0.243
Exercise habit, n (%)	29 (25%)	24 (23%)	5 (45%)	0.104
Welfare recipient, n (%)	9 (7%)	7 (6%)	2 (18%)	0.179
Occupation, n (%)				
Unemployed	45 (39%)	38 (36%)	7 (63%)	0.402
Homemaker	22 (19%)	21 (20%)	1 (9%)	
Student	2 (1%)	2 (1%)	0 (0%)	
Part-time job	10 (8%)	10 (9%)	0 (0%)	
Self-employment	11 (9%)	11 (10%)	0 (0%)	
Salaried worker	25 (21%)	22 (21%)	3 (27%)	
Education, n (%)				
College/university	32 (27%)	28 (26%)	4 (36%)	0.671
Vocational School	26 (22%)	25 (24%)	1 (9%)	
High school	42 (36%)	38 (36%)	4 (36%)	
Junior high school	15 (13%)	13 (12%)	2 (18%)	
Pain-NRS, points	5.5 (2.0)	5.5 (2.0)	5.6 (2.2)	0.857
Pain Catastrophizing Scale, points	34.4 (11.1)	34.2 (11.2)	36.0 (10.6)	0.630
Rumination, points	12.3 (3.5)	12.3 (3.6)	12.7 (3.3)	0.718
Magnification, points	6.8 (3.2)	6.7 (3.3)	7.5 (3.0)	0.443
Helplessness, points	15.2 (5.4)	15.2 (5.4)	15.7 (5.6)	0.770
HADS Anxiety, points	8.4 (4.7)	8.4 (4.8)	7.9 (3.3)	0.704
HADS Depression, points	8.7 (5.0)	8.8 (5.1)	8.0 (4.4)	0.604
PDAS, points	24.0 (12.5)	23.2 (11.9)	32.0 (15.0)	0.026*
PSEQ, points	26.9 (15.2)	26.9 (15.0)	26.0 (17.7)	0.853
Athens Insomnia Scale, points	9.5 (5.4)	9.6 (5.5)	9.1 (4.1)	0.797
Locomo-25, points	36.4 (22.2)	35.9 (22.4)	40.7 (20.9)	0.500
EQ-5D-3L,points	0.56 (0.17)	0.56 (0.16)	0.51 (0.20)	0.354

EQ-5D-3L, Euro Quality of life-5 Dimensions-3 level; HADS, Hospital Anxiety and Depression Scale; Locomo-25, the 25-question Geriatric Locomotive Function Scale; Maudsley L-score, lying tendencies score; NRS, Numerical Rating Scale; PDAS, Pain Disability Assessment Scale; PSEQ, Pain Self-Efficacy Questionnaire. Data from continuous variables are shown as mean (standard deviation). Data from categorical variables are shown as number (%). *Significance level was set at < 5% between Low L-score ≤ 25 points and High L-score > 25 points.

Correlations between the Maudsley subscores and variables are shown in **Table 2**. The Maudsley N-score was significantly associated with Pain Catastrophizing Scale (r = 0.370), HADS Anxiety (r = 0.491), HADS Depression (r = 0.452), PSEQ (r = −0.283), Athens Insomnia Scale (r = 0.292), and EQ-5D-3L (r = −0.186). The Maudsley E-score was significantly associated with Pain Catastrophizing Scale (r = −0.218). The Maudsley L-score was significantly associated with age (r = 0.274). The correlations are similar in patients with frequent lying (Maudsley L-score, > 25 points; n = 11) (S2 Table) and those with normal (Maudsley L-score, ≤ 25 points; n = 104) (S3 Table). Thus, we used the data from all patients for the subsequent analysis (n = 115).

**Table 2:** Correlation between Maudsley subscore and the variables in overall patients.

	Maudsley E-score		Maudsley N-score		Maudsley L-score	
	Correlation coefficient	p-value	Correlation coefficient	p-value	Correlation coefficient	p-value
Age, year	0.097	0.300	−0.092	0.326	**0.274**	0.003*
Height, cm	0.077	0.413	0.033	0.725	−0.170	0.068
Weight, kg	0.140	0.133	0.062	0.509	−0.164	0.079
Body mass index, kg/m^2^	0.141	0.130	0.060	0.518	−0.099	0.292
Pain-NRS, points	0.131	0.160	0.042	0.654	0.164	0.078
Pain Catastrophizing Scale, points	**−0.218**	0.019*	**0.370**	<0.001*	−0.097	0.304
Rumination, points	**−0.216**	0.020*	**0.377**	<0.001*	−0.112	0.233
Magnification, points	**−0.195**	0.036*	**0.376**	<0.001*	−0.123	0.189
Helplessness, points	**−0.190**	0.042*	**0.286**	0.001*	−0.051	0.590
HADS Anxiety, points	−0.123	0.190	**0.491**	<0.001*	−0.136	0.147
HADS Depression, points	−0.167	0.073	**0.452**	<0.001*	−0.126	0.180
PDAS	0.034	0.714	0.050	0.589	0.142	0.128
PSEQ	0.175	0.061	**−0.283**	0.002*	0.071	0.449
Athens Insomnia Scale, points	−0.052	0.578	**0.292**	0.001*	−0.151	0.107
Locomo-25, points	0.047	0.616	0.163	0.081	0.058	0.537
EQ-5D-3L,points	0.005	0.955	**−0.186**	0.046*	−0.041	0.666

EQ-5D-3L, Euro Quality of life-5 Dimensions-3 level; HADS, Hospital Anxiety and Depression Scale; Locomo-25, the 25-question Geriatric Locomotive Function Scale; Maudsley E-score, introversion/extroversion score; Maudsley L-score, lying tendencies score; Maudsley N-score, neurotic tendency score; NRS, Numerical Rating Scale; PDAS, Pain Disability Assessment Scale; PSEQ, Pain Self-Efficacy Questionnaire. * Significance level was set at < 5% by the Pearson correlation coefficient test.

The patients with the neurotic tendency (Maudsley N-score, > 28 points) showed significantly higher Pain Catastrophizing Scale, HADS Anxiety, HADS Depression, and Athens Insomnia Scale, while lower PSEQ, and EQ-5D-3L, compared to patients with low neurotic tendency or average (Maudsley N-score, ≤ 28 points) (Table 3). Comparison of data between patients with introversion and those with extroversion (Maudsley E-score) showed no statistical differences **(**Table 4).

**Table 3:** Comparison of data between patients with neurotic tendency and those with low neurotic tendency (Maudsley N-score).

	Low neurotic tendency (N-score, < 19) (n = 60)	Average (N-score, 19–28) (n = 27)	Neurotic tendency (N-score, > 28) (n = 28)	p-value
Age, year	55.6 (15.3)	57.4 (14.6)	51.1 (15.5)	0.275
Women, n (%)	37 (61%)	18 (66%)	18 (64%)	0.900
Height, cm	160.3 (8.3)	159.3 (8.2)	161.5 (8.8)	0.636
Weight, kg	58.8 (12.4)	57.6 (13.9)	61.8 (15.2)	0.489
Body mass index, kg/m^2^	22.7 (3.7)	22.6 (4.2)	23.6 (5.3)	0.618
Married, n (%)	39 (65%)	16 (59%)	16 (57%)	0.744
Exercise habit, n (%)	17 (28%)	5 (18%)	7 (25%)	0.621
Welfare recipient, n (%)	5 (8%)	1 (3%)	3 (10%)	0.612
Occupation, n (%)				
Unemployed	23 (38%)	12 (44%)	10 (35%)	0.825
Homemaker	9 (15%)	6 (22%)	7 (25%)	
Student	0 (0%)	1 (3%)	1 (3%)	
Part-time job	7 (11%)	1 (3%)	2 (7%)	
Self-employment	7 (11%)	2 (7%)	2 (7%)	
Salaried worker	14 (23%)	5 (18%)	6 (21%)	
Education, n (%)				
College/university	20 (33%)	3 (11%)	9 (32%)	0.319
Vocational School	12 (20%)	8 (29%)	6 (21%)	
High school	21 (35%)	10 (37%)	11 (39%)	
Junior high school	7 (11%)	6 (22%)	2 (7%)	
Pain-NRS, points	5.4 (2.0)	5.1 (2.1)	6.2 (1.7)	0.102
Pain Catastrophizing Scale, points	30.3 (11.6)	36.3 (10.2)†	41.3 (6.3)†	<0.001*
Rumination, points	10.9 (3.8)	13.4 (2.8)†	14.3 (1.7)†	<0.001*
Magnification, points	5.7 (3.2)	7.3 (3.1)	8.7 (2.6)†	<0.001*
Helplessness, points	13.7 (5.6)	15.5 (5.0)	18.2 (3.7)†	0.001*
HADS Anxiety, points	6.2 (4.0)	9.7 (4.2)†	11.7 (4.0)†	<0.001*
HADS Depression, points	6.8 (4.6)	9.3 (5.1)	12.3 (3.8)†‡	<0.001*
PDAS, points	22.3 (13.2)	27.0 (12.0)	24.8 (11.0)	0.265
PSEQ, points	30.3 (15.3)	27.4 (15.4)	19.1 (12.0)†	0.005*
Athens Insomnia Scale, points	8.0 (4.8)	11.1 (6.5)†	11.2 (4.4)†	0.006*
Locomo-25, points	32.3 (22.2)	38.4 (21.4)	43.1 (21.7)	0.088
EQ-5D-3L,points	0.60 (0.19)	0.52 (0.13)	0.51 (0.14)†	0.014*

EQ-5D-3L, Euro Quality of life-5 Dimensions-3 level; HADS, Hospital Anxiety and Depression Scale; Locomo-25, the 25-question Geriatric Locomotive Function Scale; Maudsley N-score, neurotic tendency score; NRS, Numerical Rating Scale; PDAS, Pain Disability Assessment Scale; PSEQ, Pain Self-Efficacy Questionnaire. Data from continuous variables are shown as mean (standard deviation). Data from categorical variables are shown as number (%). Significance level was set at < 5%. *, among groups by ANOVA test. †, versus Low neurotic tendency (N-score, < 19) by Tukey’s test. ‡, versus Average (N-score, 19–28) by Tukey’s test.

**Table 4:** Comparison of data between patients with introversion and those with extroversion (Maudsley E-score).

	Introversion (E-score, < 21) (n = 48)	Average (E-score, 21–31) (n = 34)	Extroversion (E-score, > 31) (n = 33)	p-value
Age, year	53.5 (15.1)	57.0 (15.8)	54.8 (15.2)	0.603
Women, n (%)	31 (64%)	17 (50%)	25 (75%)	0.089
Height, cm	159.6 (8.6)	161.1 (8.5)	160.6 (8.2)	0.737
Weight, kg	56.5 (12.6)	59.9 (14.1)	62.5 (13.4)	0.138
Body mass index, kg/m^2^	22.0 (3.8)	22.9 (3.8)	24.2 (4.9)	0.075
Married, n (%)	25 (52%)	24 (70%)	22 (66%)	0.186
Exercise habit, n (%)	12 (25%)	8 (23%)	9 (27%)	0.939
Welfare recipient, n (%)	3 (6%)	3 (8%)	3 (9%)	0.867
Occupation, n (%)				
Unemployed	19 (39%)	13 (38%)	13 (39%)	0.620
Homemaker	8 (16%)	4 (11%)	10 (30%)	
Student	1 (2%)	0 (0%)	1 (3%)	
Part-time job	6 (12%)	3 (8%)	1 (3%)	
Self-employment	4 (8%)	4 (11%)	3 (9%)	
Salaried worker	10 (20%)	10 (29%)	5 (15%)	
Education, n (%)				
College/university	16 (33%)	10 (29%)	6 (18%)	0.746
Vocational School	9 (18%)	9 (26%)	8 (24%)	
High school	17 (35%)	12 (35%)	13 (39%)	
Junior high school	6 (12%)	3 (8%)	6 (18%)	
Pain-NRS, points	5.2 (1.8)	5.6 (1.9)	5.8 (2.3)	0.323
Pain Catastrophizing Scale, points	37.1 (8.2)	32.5 (11.7)	32.4 (13.6)	0.093
Rumination, points	13.3 (2.5)	11.7 (3.8)	11.6 (4.1)	0.050
Magnification, points	7.4 (2.7)	6.4 (3.4)	6.2 (3.7)	0.187
Helplessness, points	16.3 (4.1)	14.4 (5.7)	14.5 (6.5)	0.207
HADS Anxiety, points	9.1 (4.4)	7.1 (3.8)	8.7 (5.6)	0.142
HADS Depression, points	9.7 (4.7)	7.3 (4.6)	8.7 (5.7)	0.097
PDAS, points	24.8 (12.3)	21.5 (11.6)	25.4 (13.5)	0.367
PSEQ, points	23.5 (13.6)	30.7 (16.0)	27.7 (15.8)	0.099
Athens Insomnia Scale, points	9.9 (5.4)	8.8 (5.9)	9.9 (4.8)	0.626
Locomo-25, points	36.6 (22.4)	32.0 (19.4)	40.5 (24.2)	0.293
EQ-5D-3L,points	0.54 (0.16)	0.61 (0.14)	0.53 (0.19)	0.080

EQ-5D-3L, Euro Quality of life-5 Dimensions-3 level; HADS, Hospital Anxiety and Depression Scale; Locomo-25, the 25-question Geriatric Locomotive Function Scale; Maudsley E-score, introversion/extroversion score; NRS, Numerical Rating Scale; PDAS, Pain Disability Assessment Scale; PSEQ, Pain Self-Efficacy Questionnaire. Data from continuous variables are shown as mean (standard deviation). Data from categorical variables are shown as number (%). Significance level was set at < 5%. *, among groups by ANOVA test. †, versus Introversion (E-score, < 21) by Tukey’s test. ‡, versus Average (E-score, 21–31) by Tukey’s test.

**Fig 1:**
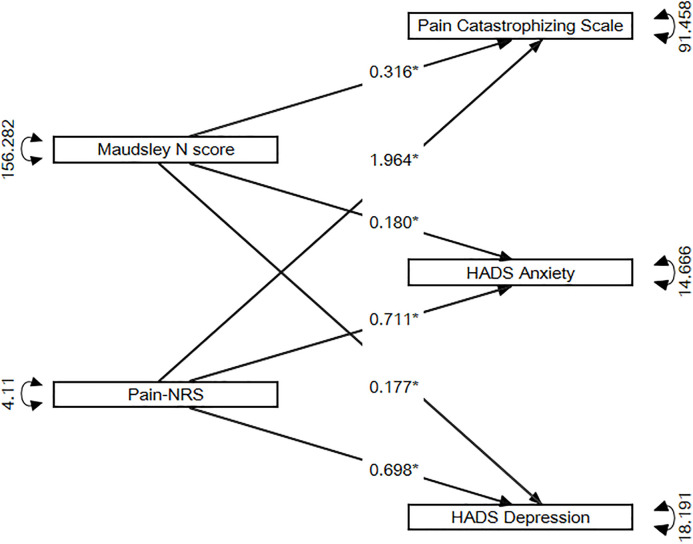
Path analysis of the Maudsley N-score and Pain-NRS. HADS, Hospital Anxiety and Depression Scale; Maudsley N-score, neurotic tendency score; NRS, Numerical Rating Scale. Maudsley N-score and pain-NRS significantly influenced with Pain Catastrophizing Scale, HADS Anxiety, and HADS Depression in the path analysis. However, the unstandardized coefficients for Maudsley N-score was small, compared to pain-NRS in each outcome. The fitness of the model was poor. *Significance level was set at < 5%.

The path analysis included the outcomes with moderate correlation to improve the fitness of the model. Maudsley N-score and pain-NRS significantly influenced with Pain Catastrophizing Scale, HADS Anxiety, and HADS Depression in the path analysis. However, the unstandardized coefficients for Maudsley N-score was small (0.177–0.316), compared to pain-NRS (0.698–1.964) in each outcome. The fitness of the model was poor (GFI, 0.686; AGFI, 0.566; CFI, 0.468; TLI, 0.329; and RMSEA, 0.531) (Fig 1) (Table 5).

**Table 5:** Unstandardized coefficients, S.E., and 95% Confidence Intervals for the model with path analysis of the Maudsley N-score and Pain-NRS.

Effects			Unstandardized coefficient	S.E.	95% Confidence Interval	p-value
Maudsley N-score	→	Pain Catastrophizing Scale	0.316	0.071	0.176–0.456	<0.001*
Pain-NRS	→	Pain Catastrophizing Scale	1.964	0.440	1.101–2.827	<0.001*
Maudsley N-score	→	HADS Anxiety	0.180	0.029	0.124–0.236	<0.001*
Pain-NRS	→	HADS Anxiety	0.711	0.176	0.365–1.056	<0.001*
Maudsley N-score	→	HADS Depression	0.177	0.032	0.115–0.240	<0.001*
Pain-NRS	→	HADS Depression	0.698	0.196	0.313–1.083	<0.001*

HADS, Hospital Anxiety and Depression Scale; Maudsley N-score, neurotic tendency score; NRS, Numerical Rating Scale; S.E., standard error. *Significance level was set at < 5%.

## Discussion

The present study demonstrated that patients with the neurotic tendency showed significantly higher levels of pain catastrophizing, anxiety, and depression, compared with patients with low or average levels of neurotic tendency among individuals with CLBP, with small effect sizes.

Neuroticism is the propensity to experience negative emotions, including anxiety, fear, sadness, anger, guilt, disgust, irritability, loneliness, worry, self-consciousness, dissatisfaction, hostility, embarrassment, reduced self-confidence, and feelings of vulnerability, in response to various types of stress [5]. Negative life experiences, whether in aggregate or in particular, are generally associated with increases in neuroticism [39]. Neuroticism is assessed using a variety of standardized questionnaire instruments [5]. There is a common tendency to equate personality among patients [16]. Although personality traits are not all-encompassing, nor do they adequately capture the units of analysis typically included under the broad umbrella of personality psychology [16]. Neuroticism is correlated with brain activity in regions involved in emotional and cognitive pain processing, including the para-hippocampus, insula, thalamus, and anterior cingulate cortex [40]. Whole-brain resting-state functional neuroimaging studies suggested neuroticism is positively correlated with the brain activity in the left middle temporal gyrus, left striatum, and right hippocampus, and negatively correlated in the left superior temporal gyrus, and right supramarginal gyrus [41]. Several potential mechanisms have been proposed to explain the relationship between neuroticism and chronic pain through heightened physical sensitivity, pain-related beliefs, emotional distress, less-adaptive, or passive coping strategies, however, these mechanisms are complex and remain uncertain [8]. Personality vulnerabilities may lead certain individuals to be more threatened by pain, thereby resulting in greater pain-related fear, avoidance and disability [42]. Meanwhile, evidences whether neuroticism is associated with a greater pain-related psychological disturbance [43], or not [44] are contradictory. The present study suggested that neuroticism has a statistically significant, but small, effect on pain-related psychological variables.

Neuroticism and other personality traits were found to be modifiable through both clinical and nonclinical interventions [45]. Among personality domains, neuroticism and extraversion show the greatest chance of change [45]. Numerous studies reported the effects of cognitive behavior therapy, psychodynamic approaches, and nonclinical intervention, however, the type of therapy employed is not strongly associated with the magnitude of change in personality traits [45]. Examination of the relationship between treatment duration and personality change showed that most gains occur within the first month of therapy [45]. A variety of efforts at improving cognitive functioning could change personality traits more rapidly than commonly thought [45]. Personality traits could be useful in guiding clinical decision making for rehabilitation [10]. That is, high levels of neuroticism could be associated with worse prognosis and low adherence with a rehabilitation program [10]. The patient may benefit from more frequent in-clinic visits and additional psychological intervention.

A previous study reported the characteristics and pain in the individuals with high Maudsley L-score [21]. Participants were recruited regardless of disease or pain, and three quarter reported no pain or only mild pain [21]. Based on these results, they suggested that the Maudsley L-score is not strongly influenced by an individual’s state of mind or situation [21]. The Maudsley L-score was significantly higher among individuals with a high degree of pain [21]. Similarly, the present study suggested Maudsley L-score showed no concordant relationship with any pain-related variables. The present study included patients with CLBP from outpatient clinic, who reported an average of 5.5 on a 0–10 point pain-NRS. In both studies, fewer than 10% of participants exhibited a high Maudsley L-score. The Maudsley Personality Inventory easily measures personality, comparable to the Minnesota Multiphasic Personality Inventory, and includes a lie scale. PDAS scores were higher in patients with high Maudsley L-scores, although this interpretation should be made with caution due to the small sample size.

Chronic postsurgical or posttraumatic pain is defined that develops or increases in intensity following a surgical procedure or tissue injury and persists beyond the normal healing process [46,47]. Patients with chronic secondary pain indicated a higher diagnostic fit for new diagnoses compared with those with chronic primary pain [48]. Stigma experienced by individuals with chronic pain affects multiple aspects of daily life [49]. Distinguish between primary nonspecific or secondary pain would result in an accurate statistical representation, and lead to the development of effective treatments for patients [46,47].

There are several limitations to the present study. First, personality traits were measured using self-reported questionnaire. Most tools for personality traits are concerned about potential oversimplification and response biases [45]. Second, the present study used a cross-sectional design conducted in a single country across a couple of medical centers. Our observations must therefore be interpreted with caution.

## Conclusions

The patients with the neurotic tendency showed significantly higher pain catastrophizing, anxiety, and depression, compared to patients with low neurotic tendency or average. The Maudsley N-score influenced with pain-related psychological variables in CLBP, but the impact was small.

## Supporting information

S1 Table A) Comparison among Maudsley subscores. B) Correlation between Maudsley subscores. Maudsley E-score, introversion/extroversion score; Maudsley L-score, lying tendencies score; Maudsley N-score, neurotic tendency score. Data from continuous variables are shown as mean (standard deviation). Data from categorical variables are shown as number (%). Significance level was set at < 5%. *, among groups by ANOVA test. †, versus low group by Tukey’s test. ‡, versus average group by Tukey’s test.
(DOCX)

S2 Table Correlation between Maudsley subscore and the variables in patients with Frequent lying in Maudsley L-score (n = 11). EQ-5D-3L, Euro Quality of life-5 Dimensions-3 level; HADS, Hospital Anxiety and Depression Scale; Locomo-25, the 25-question Geriatric Locomotive Function Scale; Maudsley E-score, introversion/extroversion score; Maudsley L-score, lying tendencies score; Maudsley N-score, neurotic tendency score; NRS, Numerical Rating Scale; PDAS, Pain Disability Assessment Scale; PSEQ, Pain Self-Efficacy Questionnaire. * Significance level was set at < 5% by the Pearson correlation coefficient test.
(DOCX)

S3 Table Correlation between Maudsley subscore and the variables in patients with normal score in Maudsley L-score (n = 104). EQ-5D-3L, Euro Quality of life-5 Dimensions-3 level; HADS, Hospital Anxiety and Depression Scale; Locomo-25, the 25-question Geriatric Locomotive Function Scale; Maudsley E-score, introversion/extroversion score; Maudsley L-score, lying tendencies score; Maudsley N-score, neurotic tendency score; NRS, Numerical Rating Scale; PDAS, Pain Disability Assessment Scale; PSEQ, Pain Self-Efficacy Questionnaire. * Significance level was set at < 5% by the Pearson correlation coefficient test.
(DOCX)
