# Supplementary material for: Personality traits have an effect on pain-related psychological variables in patients with chronic low back pain
Source: PLoS One. 2026 Jul 31;21(7):e0354827. doi: 10.1371/journal.pone.0354827 (PMC13426982; doi:10.1371/journal.pone.0354827)
Supplement: S1 Table — B) Correlation between Maudsley subscores. Maudsley E-score, introversion/extroversion score; Maudsley L-score, lying tendencies score; Maudsley N-score, neurotic tendency score. Data from continuous variables are shown as mean (standard deviation). Data from categorical variables are shown as number (%). Significance level was set at < 5%. *, among groups by ANOVA test. †, versus low group by Tukey’s test. ‡, versus average group by Tukey’s test. (DOCX) [file pone.0354827.s001.docx]

**Supplemental Table 1.** A) Comparison among Maudsley subscores

|  | Overall  (n=115) | Low L-score  ≤ 25 points  (n=104) | High L-score  > 25 points  (n=11) | p-value | Low neurotic tendency  (N-score,  < 19) (n=60) | Average  (N-score,  19–28) (n=27) | Neurotic tendency  (N-score,  > 28) (n=28) | p-value |
| --- | --- | --- | --- | --- | --- | --- | --- | --- |
| Maudsley E-score, points | 23.8 (11.8) | 23.6 (11.8) | 26.0 (12.3) | 0.518 | 26.5 (10.9) | 23.0 (12.2) | 18.9 (12.0)† | 0.016* |
| Introversion (< 21), n (%) | 48 (41 %) | 43 (41 %) | 5 (45 %) | 0.965 | 19 (31 %) | 11 (40 %) | 18 (64 %) | 0.044* |
| Average (21–31), n (%) | 34 (29 %) | 31 (29 %) | 3 (27 %) |  | 22 (36 %) | 9 (33 %) | 3 (10 %) |  |
| Extroversion (> 31), n (%) | 33 (28 %) | 30 (28 %) | 3 (27 %) |  | 19 (31 %) | 7 (25 %) | 7 (25 %) |  |
| Maudsley N-score, points | 18.4 (12.5) | 19.3 (12.6) | 9.7 (8.1) | 0.015* | 8.0 (4.9) | 23.3 (2.8)† | 36.0 (4.0)†‡ | <0.001* |
| Low neurotic tendency (< 19), n (%) | 60 (52 %) | 51 (49 %) | 9 (81 %) | 0.076 | 60 (100 %) | 0 (0 %) | 0 (0 %) |  |
| Average (19–28), n (%) | 27 (23 %) | 25 (24 %) | 2 (18 %) |  | 0 (0 %) | 27 (100 %) | 0 (0 %) |  |
| Neurotic tendency (> 28), n (%) | 28 (24 %) | 28 (26 %) | 0 (0 %) |  | 0 (0 %) | 0 (0 %) | 28 (100 %) |  |
| Maudsley L-score, points | 17.1 (6.4) | 15.9 (5.6) | 27.8 (2.0) | <0.001* | 19.4 (5.9) | 15.4 (7.0)† | 13.8 (4.8)† | <0.001* |
| Normal (≤ 25), n (%) | 104 (90 %) | 104 (100 %) | 0 (0 %) |  | 51 (85 %) | 25 (92 %) | 28 (100 %) | 0.076 |
| Frequent lying (> 25), n (%) | 11 (9 %) | 0 (0 %) | 11 (100 %) |  | 9 (15 %) | 2 (7 %) | 0 (0 %) |  |

Maudsley E-score, introversion/extroversion score; Maudsley L-score, lying tendencies score; Maudsley N-score, neurotic tendency score. Data from continuous variables are shown as mean (standard deviation). Data from categorical variables are shown as number (%). Significance level was set at < 5%. *, among groups by ANOVA test. †, versus low group by Tukey’s test. ‡, versus average group by Tukey’s test.

*Continue.*

|  | Introversion (E-score, < 21) (n=48) | Average (E-score, 21–31) (n=34) | Extroversion (E-score, > 31) (n=33) | p-value |
| --- | --- | --- | --- | --- |
| Maudsley E-score, points | 12.0 (5.0) | 26.8 (2.9)† | 37.9 (5.5)†‡ | <0.001* |
| Introversion (< 21), n (%) | 48 (100 %) | 0 (0 %) | 0 (0 %) |  |
| Average (21–31), n (%) | 0 (0 %) | 34 (100 %) | 0 (0 %) |  |
| Extroversion (> 31), n (%) | 0 (0 %) | 0 (0 %) | 33 (100 %) |  |
| Maudsley N-score, points | 22.6 (13.5) | 14.8 (9.7)† | 16.0 (12.1)† | 0.008* |
| Low neurotic tendency (< 19), n (%) | 19 (39 %) | 22 (64 %) | 19 (57 %) | 0.044* |
| Average (19–28), n (%) | 11 (22 %) | 9 (26 %) | 7 (21 %) |  |
| Neurotic tendency (> 28), n (%) | 18 (37 %) | 3 (8 %) | 7 (21 %) |  |
| Maudsley L-score, points | 15.6 (7.0) | 17.1 (6.0) | 19.3 (5.1)† | 0.039* |
| Normal (≤ 25), n (%) | 43 (89 %) | 31 (91 %) | 30 (90 %) | 0.965 |
| Frequent lying (> 25), n (%) | 5 (10 %) | 3 (8 %) | 3 (9 %) |  |

**Supplemental Table 1.** B) Correlation between Maudsley subscores

|  | Maudsley E-score | | Maudsley N-score | | Maudsley L-score | |
| --- | --- | --- | --- | --- | --- | --- |
|  | Correlation coefficient | p-value | Correlation coefficient | p-value | Correlation coefficient | p-value |
| Maudsley E-score | - | - | **-0.326** | **<0.001*** | **0.296** | **0.001*** |
| Maudsley N-score | **-0.326** | **<0.001*** | - | - | **-0.441** | **<0.001*** |
| Maudsley L-score | **0.296** | **0.001*** | **-0.441** | **<0.001*** | - | - |
