# Supplementary material for: Personality traits have an effect on pain-related psychological variables in patients with chronic low back pain
Source: PLoS One. 2026 Jul 31;21(7):e0354827. doi: 10.1371/journal.pone.0354827 (PMC13426982; doi:10.1371/journal.pone.0354827)
Supplement: S2 Table — EQ-5D-3L, Euro Quality of life-5 Dimensions-3 level; HADS, Hospital Anxiety and Depression Scale; Locomo-25, the 25-question Geriatric Locomotive Function Scale; Maudsley E-score, introversion/extroversion score; Maudsley L-score, lying tendencies score; Maudsley N-score, neurotic tendency score; NRS, Numerical Rating Scale; PDAS, Pain Disability Assessment Scale; PSEQ, Pain Self-Efficacy Questionnaire. * Significance level was set at < 5% by the Pearson correlation coefficient test. (DOCX) [file pone.0354827.s002.docx]

**Supplemental Table 2.** Correlation between Maudsley subscore and the variables in patients with Frequent lying in Maudsley L-score (n=11)

|  | Maudsley E-score | | Maudsley N-score | | Maudsley L-score | |
| --- | --- | --- | --- | --- | --- | --- |
|  | Correlation coefficient | p-value | Correlation coefficient | p-value | Correlation coefficient | p-value |
| Age, year | 0.047 | 0.890 | -0.016 | 0.961 | -0.074 | 0.828 |
| Height, cm | -0.230 | 0.496 | -0.362 | 0.273 | -0.480 | 0.135 |
| Weight, kg | 0.403 | 0.219 | -0.038 | 0.911 | -0.129 | 0.705 |
| Body mass index, kg/m^2^ | 0.563 | 0.071 | 0.129 | 0.703 | 0.057 | 0.866 |
| Pain-NRS, points | 0.159 | 0.639 | -0.050 | 0.884 | 0.398 | 0.225 |
| PCS (points) | 0.308 | 0.356 | -0.544 | 0.083 | 0.073 | 0.830 |
| Rumination (points) | 0.258 | 0.442 | -0.133 | 0.697 | 0.139 | 0.682 |
| Magnification (points) | 0.311 | 0.351 | -0.506 | 0.112 | -0.192 | 0.572 |
| Helplessness (points) | 0.261 | 0.437 | **-0.673** | **0.023*** | 0.158 | 0.640 |
| HADS Anxiety, points | 0.104 | 0.759 | 0.1281 | 0.707 | 0.453 | 0.161 |
| HADS Depression, points | 0.216 | 0.523 | -0.207 | 0.541 | 0.385 | 0.241 |
| PDAS | -0.066 | 0.846 | 0.025 | 0.941 | 0.329 | 0.322 |
| PSEQ | 0.097 | 0.776 | 0.014 | 0.965 | -0.245 | 0.468 |
| Athens Insomnia Scale, points | 0.259 | 0.440 | 0.001 | 0.996 | -0.206 | 0.542 |
| Locomo-25, points | 0.028 | 0.933 | 0.129 | 0.704 | 0.137 | 0.687 |
| EQ-5D-3L,points | 0.277 | 0.409 | 0.211 | 0.532 | -0.194 | 0.568 |
| Maudsley E-score | - | - | -0.313 | 0.348 | 0.008 | 0.979 |
| Maudsley N-score | -0.313 | 0.348 | - | - | 0.309 | 0.353 |
| Maudsley L-score | 0.008 | 0.979 | 0.309 | 0.353 | - | - |

EQ-5D-3L, Euro Quality of life-5 Dimensions-3 level; HADS, Hospital Anxiety and Depression Scale; Locomo-25, the 25-question Geriatric Locomotive Function Scale; Maudsley E-score, introversion/extroversion score; Maudsley L-score, lying tendencies score; Maudsley N-score, neurotic tendency score; NRS, Numerical Rating Scale; PDAS, Pain Disability Assessment Scale; PSEQ, Pain Self-Efficacy Questionnaire. * Significance level was set at < 5% by the Pearson correlation coefficient test.
