# Supplementary material for: Personality traits have an effect on pain-related psychological variables in patients with chronic low back pain
Source: PLoS One. 2026 Jul 31;21(7):e0354827. doi: 10.1371/journal.pone.0354827 (PMC13426982; doi:10.1371/journal.pone.0354827)
Supplement: S3 Table — EQ-5D-3L, Euro Quality of life-5 Dimensions-3 level; HADS, Hospital Anxiety and Depression Scale; Locomo-25, the 25-question Geriatric Locomotive Function Scale; Maudsley E-score, introversion/extroversion score; Maudsley L-score, lying tendencies score; Maudsley N-score, neurotic tendency score; NRS, Numerical Rating Scale; PDAS, Pain Disability Assessment Scale; PSEQ, Pain Self-Efficacy Questionnaire. * Significance level was set at < 5% by the Pearson correlation coefficient test. (DOCX) [file pone.0354827.s003.docx]

**Supplemental Table 3.** Correlation between Maudsley subscore and the variables in patients with normal score in Maudsley L-score (n=104)

|  | Maudsley E-score | | Maudsley N-score | | Maudsley L-score | |
| --- | --- | --- | --- | --- | --- | --- |
|  | Correlation coefficient | p-value | Correlation coefficient | p-value | Correlation coefficient | p-value |
| Age, year | 0.100 | 0.311 | -0.091 | 0.359 | **0.321** | **<0.001*** |
| Height, cm | 0.109 | 0.268 | 0.016 | 0.864 | -0.104 | 0.293 |
| Weight, kg | 0.126 | 0.202 | 0.050 | 0.613 | -0.147 | 0.135 |
| Body mass index, kg/m^2^ | 0.103 | 0.296 | 0.055 | 0.574 | -0.117 | 0.236 |
| Pain-NRS, points | 0.127 | 0.196 | 0.054 | 0.582 | 0.1821 | 0.064 |
| PCS (points) | **-0.273** | **0.005*** | **0.447** | **<0.001*** | -0.154 | 0.117 |
| Rumination (points) | **-0.264** | **0.006*** | **0.428** | **<0.001*** | -0.168 | 0.089 |
| Magnification (points) | **-0.248** | **0.011*** | **0.458** | **<0.001*** | **-0.197** | **0.044*** |
| Helplessness (points) | **-0.240** | **0.014*** | **0.366** | **<0.001*** | -0.089 | 0.369 |
| HADS Anxiety, points | -0.137 | 0.164 | **0.512** | **<0.001*** | -0.154 | 0.118 |
| HADS Depression, points | **-0.199** | **0.043*** | **0.490** | **<0.001*** | -0.135 | 0.170 |
| PDAS | 0.033 | 0.734 | 0.110 | 0.264 | 0.024 | 0.804 |
| PSEQ | 0.186 | 0.058 | **-0.322** | **<0.001*** | 0.113 | 0.251 |
| Athens Insomnia Scale, points | -0.075 | 0.449 | **0.308** | **0.001*** | -0.164 | 0.095 |
| Locomo-25, points | 0.0449 | 0.6511 | 0.1865 | 0.058 | 0.0249 | 0.802 |
| EQ-5D-3L,points | -0.023 | 0.8177 | **-0.249** | **0.010*** | 0.0173 | 0.861 |
| Maudsley E-score | - | - | **-0.324** | **<0.001*** | **0.332** | **<0.001*** |
| Maudsley N-score | **-0.324** | **<0.001*** | - | - | **-0.407** | **<0.001*** |
| Maudsley L-score | **0.332** | **<0.001*** | **-0.407** | **<0.001*** | - | - |

EQ-5D-3L, Euro Quality of life-5 Dimensions-3 level; HADS, Hospital Anxiety and Depression Scale; Locomo-25, the 25-question Geriatric Locomotive Function Scale; Maudsley E-score, introversion/extroversion score; Maudsley L-score, lying tendencies score; Maudsley N-score, neurotic tendency score; NRS, Numerical Rating Scale; PDAS, Pain Disability Assessment Scale; PSEQ, Pain Self-Efficacy Questionnaire. * Significance level was set at < 5% by the Pearson correlation coefficient test.
